# Increasing rates of ulnar collateral ligament repair outpace reconstruction in isolated injuries: review of a Texas surgical database

**DOI:** 10.1016/j.jseint.2022.10.008

**Published:** 2022-11-08

**Authors:** Taylor J. Willenbring, Eden C. Epner, Ryan J. Warth, James M. Gregory

**Affiliations:** a State University of New York Upstate Medical University, Syracuse, NY, USA; b University of Texas Health Science Center at Houston, Houston, TX, USA

**Keywords:** Ulnar collateral ligament, Elbow, Ligament reconstruction, Ligament repair, Evidence-based medicine, Surgical indications

## Abstract

**Background:**

The gold standard of treatment for ulnar collateral ligament (UCL) injuries has been reconstruction. Despite early repair studies yielding less than satisfactory results, there has been recent renewed interest in UCL repair due to improved outcomes and new technologies. Data regarding clinical use of these procedures are lacking. The purpose of this study was to define the epidemiological trends of UCL repair and reconstruction surgery from 2010 to 2019, compare demographic characteristics of patients undergoing either procedure, and determine incidence of concomitant procedures in each surgical group as well as comparing respective patient-level charges.

**Methods:**

A retrospective database analysis of UCL surgeries was performed through the Texas Healthcare Information Collection database, a comprehensive and publicly available statewide billing dataset. Inclusion criteria were defined using Current Procedural Terminology billing codes for elbow UCL repair and reconstruction between 2010 through 2019, excluding patients who had concomitant elbow fractures or lateral collateral ligament tears indicative of high-energy trauma. Procedural volume changes, patient demographics, and commonly performed concomitant procedures including elbow arthroscopy, ulnar nerve surgery, and platelet-rich plasma injection were compared. Total patient-level charges were compared across groups.

**Results:**

A total of 1664 patients were included, consisting of 484 UCL repairs and 1180 reconstructions. Total UCL surgeries increased eleven-fold when corrected for population growth from 2010 (N = 25) to 2019 (N = 315). In 2010, repair constituted 23% of all UCL tear surgeries and increased to 40% by the end of 2019. The annual frequency of UCL repair increased at a 5.4% faster rate than UCL reconstruction from 2010 to 2019 (*P* < .001). There were no significant differences between any demographic data between UCL repair and reconstruction except for rural surgical settings which demonstrated 1.8 times greater odds of undergoing reconstruction (*P* = .05). There were no differences among commonly associated procedures including ulnar nerve surgery (*P* = .217), elbow arthroscopy (*P* = .092), and platelet-rich plasma injection (*P* = .837) with no differences in patient-level charges at any time point (*P* = .47).

**Conclusion:**

While reconstruction remains more common, the annual frequency of UCL repair is increasing at a faster rate. Since were no demographic differences aside from surgical setting, it can be inferred that patients who were previously receiving reconstruction are instead undergoing repair. This highlights the need for future studies to further identify surgical indications for the two interventions.

Ulnar collateral ligament (UCL) injury is one of the most common orthopedic injuries among overhead athletes.^2^^,^^19^ Because the UCL plays a vital role in the valgus stability of the elbow joint, persons who subject their medial elbows to repeated high speed valgus torque are especially susceptible to UCL injury or tear. If conservative measures such as physical therapy regimens and other nonoperative treatments fail in the setting of UCL injury, operative approaches should be considered, especially for high-level overhead athletes with complete UCL tears.^2^ Since partial or complete UCL tears can be potentially career-ending injuries, high-level athletes and their healthcare teams seek successful and timely return-to-play with the least invasive approach possible. In recent decades, there has been an overall rise in both UCL repair and reconstruction.^2^^,^^15^^,^^16^

Historically, UCL reconstruction was first performed in 1974 by Dr. Frank Jobe on the professional baseball pitcher Tommy John and was first published in 1986.^14^ Around the same time, attempts at UCL repair yielded “less than satisfactory” results with one small cohort demonstrating a 50% return to sport (RTS) with only two of the seven patients remaining in the major league (27%).^4^^,^^7^ This RTS statistic was significantly worse than Jobe’s initial cohort which demonstrated a 75% (12 of 16) RTS rate at the same level among professional players who underwent reconstruction among other contemporary studies.^1^^,^^4^ In the subsequent decades, frequency of the ‘Tommy John’ surgery on both recreational and professional athletes has increased significantly, with UCL reconstruction remaining the preferred surgical treatment of UCL tears.^9^^,^^13^^,^^15^^,^^17^

However, recent evidence from 2015 to 2022 has arisen showing improved outcomes with modified repair techniques, particularly in high-grade partial tears as well as proximal or distal tears.^3^^,^^7^^,^^8^^,^^18^^,^^20^ There has also renewed interest in repair techniques and industry technology as the understanding of elbow biomechanics has improved.^5^^,^^7^^,^^8^^,^^18^^,^^21^ Although there has been a shift in the literature paradigm, it is unknown whether there has been a significant shift in practice as well. Previous epidemiological studies examining UCL surgery have focused only on trends in UCL reconstruction.^9^^,^^13^ The prevalence of UCL repair remains unknown.

Additionally, although multiple existing studies have proposed indications for surgical versus conservative treatment, it remains unknown whether there are any differences between the surgical intervention populations.^3^^,^^6^^,^^10^^,^^11^ The purpose of this study was to investigate the practice trends for surgical treatment of UCL injuries in the state of Texas between the years 2010 through 2019 with consideration of procedure volume, patient demographics, concomitant surgical procedures, and patient-level charges. We hypothesized that the overall incidence of UCL repair will have increased more significantly than that of UCL reconstruction between 2010 and 2019 in the state of Texas, and that there would be no demographic or charge differences between the two cohorts.

## Methods

### Procedural volume

This study is a retrospective cross-sectional analysis of UCL repair, reconstruction, and any associated procedures. Data were obtained through the publicly available Texas Healthcare Information Collection (THCIC) database, which includes deidentified records of all patients discharged from eligible hospitals and ambulatory surgical centers. Since 2010, policy requires all surgical sites to report an operative case summary that includes patient demographic information, diagnostic billing codes, and a standardized list of itemized charges. Other information collected includes surgery center location, categorization of procedural setting, and type of payor for the procedure. Ethical committee approval was not required due to the anonymized nature of the database. While limited to the state of Texas, this database represents a significant population in a more thorough manner than many national databases by including all payor statuses such as self-pay and worker’s compensation. Thus, by not limiting the patient population to those covered by private or federal insurance, this study can provide insight into the comprehensive practice patterns of a large region while simultaneously offering a holistic analysis of patient charges. Of note, both Major League Baseball and Minor League Baseball players are covered by worker’s compensation, as indicated by their respective Uniform Players Contracts. The presence of this payor source in the database extends the accessible records to include one of the most high-risk patient populations. Overall incidence of UCL surgery was calculated using the 2010 and 2020 decennial census data collected by US Census Bureau.^12^

Inclusion criteria were defined using Current Procedural Terminology (CPT) codes as published by the American Medical Association. CPT codes provide a standardized method for procedural billing in the United States and allow for retrospective analysis of large populations of surgical patients. The THCIC database was queried for patients who received either UCL reconstruction (CPT 24346; N = 1255) or repair (CPT 24345; N = 514) beginning in 2010 through 2019. Exclusion criteria were defined as the presence of the respective CPT, International Classification of Diseases-9, or International Classification of Diseases-10 codes for post-traumatic indications, such as fracture (CPT 24685, N = 18) or lateral collateral ligament injury (CPT 24343 and 24344, N = 70 and 24, respectively). Changes in procedural frequency were analyzed with weighted linear regression models.

### Demographics, charges, and associated procedures

Demographic data included patient gender, age (categorized as <18, 18-44, 45-64, and >65 years), and ethnicity. Procedural setting was defined in a categorical manner as either a teaching or nonteaching hospital, depending on membership of the Council of Teaching Hospitals and Health Systems. Payor status was defined as private insurance, Medicare, Medicaid, worker’s compensation, self-pay, or other. Charges regardless of payor status were compared between repair and reconstruction for the entire timeframe as well as within each individual year. All included records were then analyzed for commonly performed concomitant procedures including surgery of the ulnar nerve (CPT 64718), platelet-rich plasma (PRP) injection (CPT 0232T), or any arthroscopic procedure of the elbow (CPT 29834, 29837, 29838).

### Literature query

Due to a subjective increase in academic interest in UCL repair, a review of literature published during the study period was performed to assess for any temporal correlation between relevant publication frequency and procedural volume. The search was performed on August 26, 2021, using the following two databases: PubMed and Embase. The electronic search engine formula was defined as follows: ((ulnar collateral ligament) AND (repair)). Studies and editorials related to UCL repair published in the English language from January 1, 2010 through December 31, 2019 were eligible for inclusion. To best gauge overall academic interest, all paper qualities and levels of clinical evidence were included regardless of data contribution. Those publications relating to the UCL of the thumb were excluded. Other exclusion criteria included UCL repair in the context of trauma, fracture, fracture-dislocations, or concomitant lateral UCL surgery likely indicating a high-energy traumatic context such as a motor vehicle collision. All included records were then identified as reviews, basic science or techniques, clinical studies, and editorials. These papers were categorized by publication year and charted in a histogram format using Microsoft Excel (Excel Version 16.52; Microsoft, Redmond, WA, USA) to allow for subjective visual analysis.

### Statistical analyses

All statistical analyses were performed using SPSS version 26 (IBM Corp., Armonk, NY, USA). Comparison of frequencies between dichotomous variables was performed using Fisher exact tests. Year-over-year operative frequencies were compared using one-way ANOVA with *post hoc* Tukey tests. Nonparametric analyses were substituted when data were not normally distributed. Weighted linear regressions were performed to compare the annual rate of change in the frequency of UCL reconstruction and repair procedures from 2010 to 2019. Statistical significance level alpha was set *a priori* at 0.05.

## Results

### Procedural volume

A total of 1769 patients met inclusion criteria consisting of 1255 records for UCL reconstruction and 514 for UCL repair. A total of 105 records were identified as post-traumatic and subsequently removed from the data sample (fracture [CPT 24685, N = 18], lateral collateral ligament injury [CPT 24343, N = 70; CPT 24344, N = 24]). The remaining 1664 records consisted of 1180 unique UCL reconstruction procedures and 484 UCL repairs.

**Figure 1:**
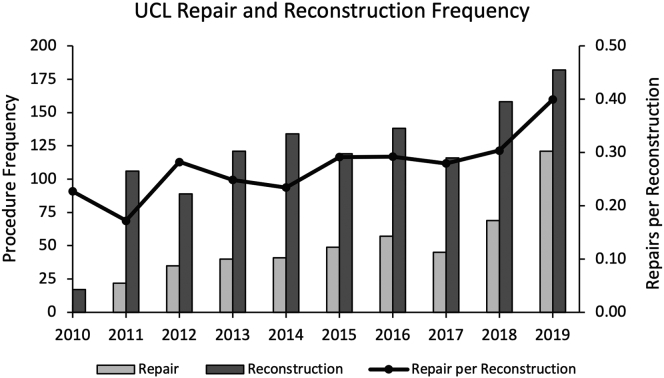
Comparison of procedural volumes of UCL repair and reconstruction from 2010 to 2019 with an overlay of the number of repairs per reconstruction indicating an increasing proportion of UCL repair. *UCL*, ulnar collateral ligament.

Total UCL surgical volume of both types increased significantly from 2010 to 2019 (N = 25 and N = 315, respectively). The overall annual incidence of UCL surgery in 2010 was 0.08 persons per 100,000 people in the state of Texas. This grew to 1.02 persons per 100,000 in 2019, an eleven-fold increase. In 2010, UCL repair constituted 23% of all UCL surgeries and steadily increased to 40% of the 315 interventions by the end of 2019 (Fig. 1). Weighted linear regression indicated that the frequency of UCL repair increased at a 5.4% faster annual rate than UCL reconstruction from 2010 to 2019 (B = 0.054 [95% confidence interval, 0.028-0.080], R^2^ = 0.70, *P* < .001).

### Demographics, charges, and associated procedures

Of all the demographic categories analyzed in this study, the most highly represented demographics were white-identifying males aged 18-44 years who underwent surgery in a metropolitan ambulatory surgical center. Most commonly, procedures were covered by private insurance. There were no significant differences between any demographic data, including patient gender, age, ethnicity, or insurance coverage for UCL repair versus reconstruction. The only notable exception existed for patients in rural surgical settings, who had 1.8 times greater odds of undergoing reconstruction (*P* = .05 [95% confidence interval, 1.0-3.3]).

A key benefit of the THCIC database is the inclusion of all payors, thus allowing a comprehensive comparison of charges for any patient who received UCL surgery. As seen in Table I, there were no differences in patient-level charges between patients receiving repair or reconstruction from 2010 through 2019 (*P* = .47), as well as any individual year within this time range.

**Table I:** Patient charge data comparisons for all repair and reconstruction for each year from 2010 through 2019, in addition to all years combined. There were no significant differences between the two groups for any time point.

Year	Repair N	Recon N	Repair charges	Reconstruction charges	*P* value
2010	5	20	$14,472.86	$12,822.86	.57
2011	22	112	$14,673.78	$17,130.04	.43
2012	38	94	$17,242.11	$17,705.39	.55
2013	43	132	$23,034.10	$20,828.88	.40
2014	44	144	$24,359.67	$23,542.49	.88
2015	57	127	$26,369.68	$23,584.91	.09
2016	60	144	$26,755.47	$29,040.98	.40
2017	49	122	$23,558.80	$28,144.00	.06
2018	74	165	$23,558.80	$28,144.00	.82
2019	124	191	$26,901.33	$28,706.84	.37
All years	516	1251	$25,103.23	$24,433.91	.47

Between UCL repair and reconstruction, there were no significant differences among commonly associated procedures, namely ulnar nerve surgery (N = 114 and N = 429, respectively; *P* = .217), elbow arthroscopy (N = 22 and N = 102, respectively; *P* = .092), and PRP injection (N = 9 and N = 29, respectively; *P* = .837). Complete demographic data and associated procedure comparisons can be seen in Table II.

**Table II:** Demographic data for all UCL reconstruction and repair from 2010 to 2019 showing likelihoods of receiving reconstruction over repair. There were no significant differences in any category except for rural surgical settings in which patients were significantly more likely to undergo reconstruction.

	Total	Repair	Reconstruction	Chi-square *P* value	Odds ratio	OR 95% CI
Procedure						
Scope	102	22	80	.092	1.521	0.937-2.468
PRP	29	9	20	.837	0.906	0.410-2.004
Ulnar nerve	429	114	315	.217	1.176	0.010-1.504
Gender						
Male	1445	417	1028	.746	1.066	0.778-1.460
Female	212	64	148	-	-	-
Race						
Hispanic	210	59	151	.808	1.045	0.757-1.441
White	1169	337	832	.813	1.032	0.818-1.300
Black	48	13	35	.872	1.104	0.579-2.105
Asian	13	6	7	.218	0.474	0.158-1.417
Native American	2	1	1	1.000	0.408	0.025-6.541
Other	429	125	304	.951	0.992	0.779-1.264
Age (y)						
<18	563	150	413	.138	1.192	0.950-1.495
18-44	995	300	695	.225	0.869	0.774-1.058
45-64	87	28	59	.554	0.853	0.538-1.356
65+	17	4	13	.791	1.331	0.432-4.103
City						
Metro	1253	366	887	.303	0.808	0.551-1.184
Micro	80	25	55	.612	0.881	0.541-1.434
Rural	76	14	62	.05∗	1.838∗	1.017-3.322∗
Payor status						
Private	1463	425	1038	.934	0.98	0.707-1.360
Medicare	19	6	13	1.000	0.884	0.334-2.339
Medicaid	46	10	36	.324	1.485	0.731-3.017
Worker's comp	90	28	62	.720	0.899	0.568-1.424
Self-pay	37	11	26	1.000	0.965	0.473-1.968
Other	7	2	5	1.000	1.021	0.197-5.282
Setting						
ASC	1100	311	789	.361	1.11	0.888-1.386
Academic	206	63	143	.623	0.917	0.668-1.260

*CI*, confidence interval; *OR*, odds ratio; *PRP*, platelet-rich plasma; *UCL*, ulnar collateral ligament.

∗ *P*≤.05

### Literature query

**Figure 2:**
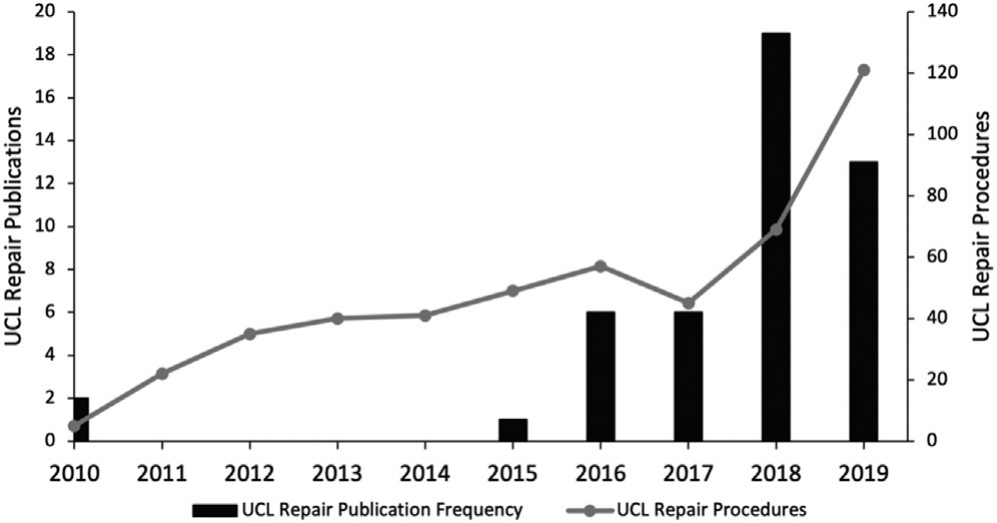
Histogram showing publications either directly studying or discussing UCL repair with overlay of repair procedural volume from 2010 through 2019. *UCL*, ulnar collateral ligament.

The initial query returned a total of 336 results, of which 60 met the inclusion criteria. After duplicate studies were removed, 47 unique publications remained that either directly studied or discussed UCL repair of the elbow from 2010 through 2019. The yearly publications ranged from zero between the years 2011 through 2014 to a maximum of 19 papers published in 2018. The overall frequency by year appears consistent with the general trend of repair procedural volume during this same period, as illustrated by overlaying repair procedural volume on the number of yearly relevant publications (Fig. 2). This trend indicates a recent increase in the academic interest in UCL repair that parallels the measured clinical pattern changes. A breakdown of publication types showed 42% of papers were basic science or technique papers, 29% were literature reviews, 26% were clinical studies, and 2% were editorials.

## Discussion

### Procedural volume and academic interest

The total interventional volume for elective UCL surgery increased significantly from 2010 through 2019, consistent with the descriptions of prior studies.^9^^,^^13^ Although both UCL repair and reconstruction increased steadily during this time, surgical repair exhibited a greater overall increase. At the beginning of the data period, repair constituted 24% of all interventions but had increased to 40% by 2019. While we cannot conclude a causal relationship to this trend, it may reflect physicians’ recognition of the improving outcomes of surgical repair over reconstruction, as noted in recent years.^7^^,^^18^ Patients who previously would have received UCL reconstruction may instead be undergoing ligament repair due to improved outcomes or perceived expanding indications.

An alternative explanation for the shift in clinical practice toward UCL repair could be recency bias in the context of a higher volume of publications discussing UCL repair in recent years. To evaluate the premise of this notion, we performed a query of the two largest databases, PubMed and Embase, to quantify the academic interest in the topic. As can be seen in Fig. 2, there remained a relative dearth of literature until 2015, after which the majority of studies from the last decade were published. This recent eruption of literature shortly precedes a distinct rise in clinical frequency of UCL repair, representing the expected correlative relationship between published literature and clinical practice. Of the 47 publications, the largest category were basic science or technique papers, which is consistent with increasing industry interest in the procedure. This of course would not explain the volume of repair which existed prior to the 2015 increase in literature production. These cases may represent the baseline interest of surgeons based on the preexisting studies.

Another consideration in driving factors behind the changing repair rates is the weighting-effect of a single busy surgical center. When analyzing a relatively uncommon procedure such as isolated UCL repair, a very busy practice could skew the generalizability to the surgeon population as a whole. In order to address this, a *post hoc* analysis was performed of the ten most productive UCL repair institutions. The busiest institution constituted 23% of all UCL repair during the study period with the second-busiest contributing 10%. When the top institution was removed from the dataset, the repair growth curve maintained the same inflections as before, reducing concern for single-group confounding.

### Demographics

Despite the increasing frequency of UCL repair and its reported outcomes, it remains unclear where it is possible to systematically direct patients toward reconstruction versus repair. Considerations for successful UCL repair are reported to include factors such as chronicity and location of the injury, quality of ligament tissue, and patient age.^20^ As a result, one may infer that UCL repair would be more prevalent in a younger cohort, who may have sustained less repetitive soft tissue trauma. However, the demographic analysis performed in this study indicated that there were no differences in patient gender, age, ethnicity, payor coverage, or total charges. It is important to note that the ages within this demographic database are grouped into categories to protect patient confidentiality. As a result, differences in mean age between the two cohorts may exist but cannot be identified precisely. However, even within the pediatric cohort (<18), which would include only patients who are not yet at a collegiate or professional level, there was no difference in surgical procedure. Surgical setting was the only relevant predictor for which procedure was performed, with rural centers 1.8 times more likely to offer UCL reconstruction. We hypothesize that these centers may be less quick to adopt newer techniques such as UCL repair.

Of the 1769 records assessed in this study, privately insured, white males aged 18 to 44 were most likely to undergo surgical treatment for UCL injury. In comparison, the 2015 study by Erickson et al, which performed a similar database study concerning exclusively UCL reconstruction from 2007 to 2011, reported that patients aged 15 to 19 were more likely to undergo UCL reconstruction than any other age group. This difference between our current study and the Erickson study may be attributed to the different populations assessed. Specifically, the Erickson study was based on a private-insurance database, in contrast with the current study, which included all payor statuses. The introduction of worker’s compensation, which represents most professional baseball players, may explain the older average age of patients undergoing UCL surgery. Thus, while the database is geographically limited, it provides the most holistic view to date of the relevant patient population.

### Associated procedures

This study analyzed three procedures commonly performed alongside UCL surgery (ulnar nerve surgery, elbow arthroscopy, and PRP injection). Due to the nature of a retrospective billing database study, it was not possible to directly identify specific pathology (eg, specific tear location or severity). We utilized these associated procedures as surrogates to provide a relative comparison of the nature of UCL injury between patients undergoing repair versus reconstruction. There were no significant differences between the two cohorts for any of the three most performed associated procedures. This suggests that concomitant injuries did not play a significant role in determining which intervention was performed.

## Limitations

This study is not without limitations. The strength of this study is limited by its retrospective nature with a primary purpose of pattern identification. Other states or regions may exhibit differing practice patterns because of cultural or economic differences than those exhibited in the state of Texas. Outcome data are not available. While the use of a billing database allows for analysis of large populations, they are necessarily limited in their detail regarding individual patient characteristics or the extent or specific location of ligament tears. Some categorizations are broadly defined. They are also subject to confounding from billing inaccuracies; however, these errors would likely have remained constant throughout the data collection period.

## Conclusion

Surgical reconstruction and repair for UCL injuries are increasingly common. From 2010 through 2019, both methods of surgical treatment increased significantly, with reconstruction remaining the most common technique comprising 60% of all surgical interventions in 2019. However, the incidence of UCL repair increased at a higher rate than that of reconstruction, reflecting its growing popularity. This growth of UCL repair over reconstruction correlates with increasing academic interest in UCL repair over recent years, during which several publications have reported more favorable outcomes than earlier studies. When comparing the patient populations undergoing both treatments, there were no significant differences regarding patient gender, age, ethnicity, or insurance coverage. Additionally, there was no difference in concomitant procedures or total charges between the two cohorts. With minimal demographic differences between the two interventions, it can be inferred that patients who previously would have undergone UCL reconstruction are now receiving repair. Future studies should seek to further clarify surgical indications for each respective intervention.

## Disclaimers

Funding: No funding was disclosed by the authors.

Conflicts of interest: The authors, their immediate families, and any research foundation with which they are affiliated have not received any financial payments or other benefits from any commercial entity related to the subject of this article.
